# Immediate and Short-Term Effects of Multimodal Neuromodulatory Rehabilitation Following Thalamotomy for Writer’s Cramp: A Case Report

**DOI:** 10.5334/tohm.1154

**Published:** 2026-06-03

**Authors:** Moritoshi Kitakami, Takashi Hoei, Seiji Etoh, Tomoko Hanada, Kentaro Kawamura, Ryosuke Hanaya, Megumi Shimodozono

**Affiliations:** 1 Division of Rehabilitation, Kagoshima University Hospital, Kagoshima, Japan; 2 Present address: Occupational Therapy Course, Department of Rehabilitation, Faculty of Allied Health Sciences, Niigata University of Rehabilitation, Murakami, Niigata, Japan; 3 Department of Rehabilitation and Physical Medicine, Kagoshima University Graduate School of Medical and Dental Sciences, Kagoshima, Japan; 4 Department of Neurosurgery, Kagoshima University Graduate School of Medical and Dental Sciences, Kagoshima, Japan

**Keywords:** writer’s cramp, thalamotomy, rehabilitation, neuromodulation, transcutaneous electrical nerve stimulation, vibration

## Abstract

**Background:**

Evidence for rehabilitation after thalamotomy for writer’s cramp is limited. We report immediate effects of multimodal rehabilitation.

**Case Report:**

A woman in her 20s with complex writer’s cramp underwent left Vo/Vim thalamotomy. Postoperatively, she completed a 6-day program combining fine motor training, transcutaneous electrical nerve stimulation, and vibration. Thalamotomy reduced dystonia (Writer’s Cramp Rating Scale [WCRS]: movement, 12 to 4; speed, 2 to 1), and rehabilitation yielded immediate writing speed gains and progressive functional recovery; although WCRS scores remained stable.

**Discussion:**

Combining bottom-up neuromodulation with task-specific training was associated with immediate gains, suggesting it may facilitate early motor relearning.

**Highlights:**

A young woman with writer’s cramp underwent a thalamotomy; followed by early multimodal rehabilitation that combined transcutaneous electrical nerve stimulation, vibratory stimulation, and fine motor training. Video-supported assessments and quantitative measures indicated reproducible within-session gains and short-term improvements in writing time, dexterity, and quality of life.

## Introduction

Writer’s cramp is a task-specific focal hand dystonia defined by involuntary co-contractions and abnormal posturing during writing [1]. Symptoms can limit activities of daily living and work performance [2]. Evidence implicates disordered sensorimotor integration within central networks [3,4]; clinically, demonstrated by a sensory trick, in which a specific peripheral sensory input can transiently normalize motor output [1]. Neurophysiological studies have indicated maladaptive motor cortical plasticity, including reduced intracortical inhibition and increased excitability [5,6], motivating therapies aimed at rebalancing these mechanisms.

Available treatments include pharmacological, non-pharmacological, and surgical options. Oral anticholinergics show limited, inconsistent benefit [2]. Botulinum neurotoxin injections are commonly used, trials have shown superiority to placebo; however, effects are temporary and may cause short-lived weakness [7]. For refractory cases, carefully selected patients may undergo stereotactic thalamotomy [8].

Rehabilitation targeting sensorimotor integration has also been used. Transcutaneous electrical nerve stimulation (TENS), vibratory stimulation, and fine motor training have supporting evidence [4,9,10]. However, whether the combination of these modalities yields synergistic benefits in the early postoperative period following thalamocortical surgery remains uncertain.

Here, we report the immediate and short-term effects of a multimodal rehabilitation program after stereotactic thalamotomy on writer’s cramp. Based on previous studies [9,10,11], we hypothesized that pairing bottom-up sensory modulation (TENS and vibratory stimulation) with top-down fine motor training might support functional gains by modulating cortical excitability and enhancing sensorimotor integration.

### Case description

**Figure 1:**
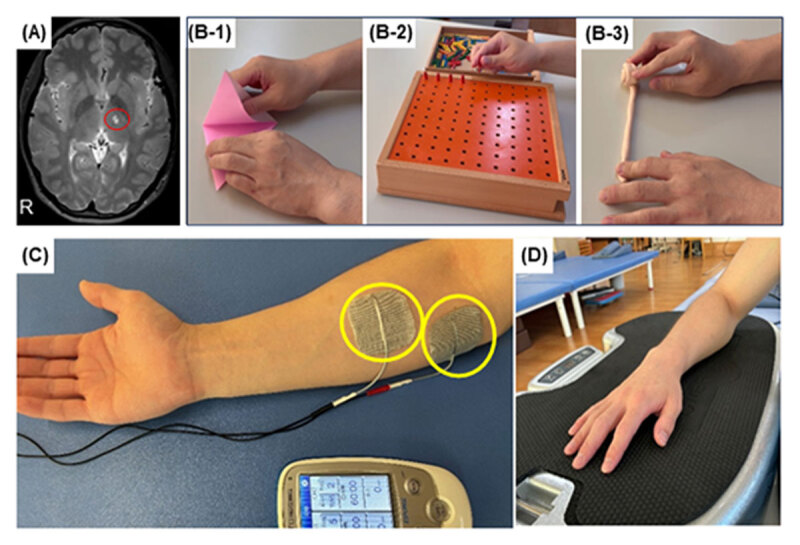
**Thalamotomy target and multimodal rehabilitation components**. **(A)** Postoperative axial T2-weighted magnetic resonance imaging showing a coagulation lesion (red circle) within the left ventro-oralis/ventral intermediate nucleus border zone following stereotactic thalamotomy. **(B)** Components of fine motor training: **(B-1)** “origami,” a Japanese paper-craft art (10 min; such as square box and crane); **(B-2)** pegboard tasks (13 min; such as single-fingertip rotation and grasp-and-hold); **(B-3)** putty exercises (7 min; including stretching and pressing, rolling). Task difficulty was progressively adjusted to the patient’s performance. **(C)** Transcutaneous electrical nerve stimulation: Surface electrodes were placed over the muscle bellies of the right flexor carpi radialis and flexor carpi ulnaris. Stimulation parameters: 50 Hz, 250 μs pulse width, and 6 mA (sensory-level intensity); device: ITO ESPURGE (ITO Co., Ltd., Kawaguchi, Japan). **(D)** Vibratory stimulation to the right upper limb: five 1-minute bouts at 35 Hz, with the right hand and distal forearm placed on a vibrating platform (Personal Power Plate; Performance Health Systems, Northbrook, IL, USA). Abbreviations: Vo, ventro-oralis nucleus; Vim, ventral intermediate nucleus.

A right-handed woman in her 20s presented with complex writer’s cramp. Symptoms began one year prior to surgery. Her symptoms were disabling and primarily triggered by writing. As a healthcare professional, she experienced excessive force on her right hand resulting in increased pen pressure, straying outside the form margins, and action tremors during patient documentation; which substantially impaired her work performance. Symptoms also interfered with other fine motor tasks, such as keyboard and smartphone operation. Sensory tricks were ineffective. Prior conservative treatments, including oral medications (diazepam, trihexyphenidyl) and rehabilitation at another facility (primarily stretching of the right upper limb and guidance on writing posture), had yielded limited benefit. Botulinum neurotoxin injections (total 50 units: 20 units to the flexor carpi ulnaris, 20 units to the flexor carpi radialis, and 10 units to the flexor digitorum superficialis [targeting the 3rd and 4th digits]) administered at our institution also provided insufficient relief. She subsequently underwent a left stereotactic thalamotomy targeting the Vo/Vim border zone at our institution (Figure 1A). Occupational therapy was initiated on postoperative day (POD) 1 and the patient was discharged on POD 7. She had no relevant medical history. This case report adhered to the Declaration of Helsinki and the CARE guidelines. The Institutional Review Board of Kagoshima University Hospital waived the requirement for ethical approval due to the retrospective nature of this single-case report.

#### Assessment

A single treating occupational therapist assessed outcomes; accordingly, the assessments were unblinded and covered both the overall progress and within-session effects. To mitigate bias, video-based evaluations were performed by rehabilitation physicians (K. K. and M. S.) who were not involved in the intervention. The observational criteria were predefined to capture clinically relevant features of dystonic handwriting: (i) the morphological integrity of stroke terminals, (ii) trajectory stability, and (iii) pen pressure. To ensure unbiased application, the video files were anonymized and presented in a randomized order; thus, the raters were blinded to the assessment time points (POD 1 vs. POD 6). They used a binary scale (presence/absence) for these features.

We assessed dystonia severity using the Writer’s Cramp Rating Scale (WCRS) [12] preoperatively (assessed by a neurosurgeon), and on POD 1 and POD 6 (assessed by an occupational therapist). Additionally, on POD 1 and POD 6, we assessed gross manual dexterity (Box and Block Test [BBT]) [13], fine finger dexterity (Nine-Hole Peg Test [NHPT]) [14], and health-related quality of life (EuroQol 5-Dimension 5-Level [EQ-5D-5L]; EuroQol Visual Analogue Scale [EQ-VAS]) [15].

To evaluate within-session effects, writing time (time to transcribe a fixed 15-character Japanese sentence in a single trial) and subjective writing satisfaction (Numerical Rating Scale [NRS], 0–10) were measured immediately before and after each of the four 40-minute sessions [16].

#### Intervention

The program comprised four 40-minute sessions on POD 1, 3, 5, and 6; and rest days on POD 2 and 4.

On POD 1 and 3, fine motor training (Figure 1B) was performed under continuous TENS. Self-adhesive electrodes were placed over the muscle bellies of the right flexor carpi radialis and flexor carpi ulnaris muscles, which exhibit task-related hypertonicity during writing. Stimulation parameters followed prior work: [9] frequency of 50 Hz, pulse width of 250 µs, and an intensity titrated to a sensory level at the beginning of each session (consistently 6 mA); to produce clear but comfortable paresthesia without visible muscle contraction (Figure 1C). The device used was the ITO ESPURGE (ITO Co., Ltd., Kawaguchi, Japan).

On POD 5 and 6, vibratory stimulation was applied in addition to TENS and fine motor training. Vibratory stimulation was administered to the right distal forearm (five 1-minute bouts) (Figure 1D). TENS was delivered continuously throughout the duration of vibratory stimulation and fine motor training. Outside formal sessions, the patient maintained a daily diary; which constituted uncontrolled exposure to handwriting.

#### Clinical course and Outcomes

Before thalamotomy, all three elements of handwriting — (i) the morphological integrity of stroke terminals, (ii) trajectory stability, and (iii) pen pressure—were severely disturbed. The preoperative video (Video 1), recorded 1 month before the thalamotomy; revealed unstable stroke trajectories, excessive flexion of the fingers and wrist, and action tremor. Preoperatively, WCRS scores indicated moderate-to-severe dystonia (Writing movement score: 12; Writing speed score: 2) (Table 1). Following thalamotomy, these scores markedly decreased on POD 1 (Writing movement score: 4; Writing speed score: 1); reflecting a reduction in dystonia severity. Quantitative assessments other than the WCRS were not conducted preoperatively because rehabilitation interventions and standardized evaluations were initiated only after surgery.

**Video 1:** **Preoperative writing performance.** Recorded approximately 1 month before thalamotomy. The video also shows the patient grasping her right forearm with her left hand to assess a sensory trick; however, no observable changes in symptoms were evident. Note: Owing to privacy masking, fine details, such as pen pressure and stroke terminals, may not be fully visible; however, clinical assessment confirmed severe disturbances.

**Table 1:** Immediate within-session effects and clinical outcomes before and after thalamotomy.


WITHIN-SESSION CHANGES (POD 1–POD 6) (FIXED 15-CHARACTER SENTENCE; SECONDS; NRS 0–10).					

POD	INTERVENTIONS	WRITING TIME, SECONDS		SATISFACTION, NRS (0–10)	
	
		PRE	POST	PRE	POST

1	① + ②	84.2	79.6	3	3

3	① + ②	79.1	69.4	3	4

5	① + ② + ③	67.6	54.3	4	6

6	① + ② + ③	63.4	48.7	4	6

**PREOPERATIVE AND POSTOPERATIVE CLINICAL MEASURES**					

**ITEM**		**PREOPERATIVE**	**INITIAL (POD 1)**	**FINAL (POD 6)**	

WCRS					

dystonic posture					

Elbow score		0	0	0	

Wrist score		2	1	1	

Finger score		3	3	3	

Latency of dystonia		2	1	1	

Writing tremor		1	0	0	

Writing movement score		12	4	4	

Writing speed score		2	1	1	

BBT, blocks					

Right hand		–	51	57	

Left hand		–	59	61	

NHPT, seconds					

Right hand		–	22.3	20.5	

Left hand		–	19.6	18.9	

EQ-5D-5L (index)		–	0.612	0.772	

EQ-VAS (0–100), points		–	40	70	

Barthel Index (0–100)		–	100	100	

Functional Independence Measure (18–126)		–	126	126	


Abbreviations: BBT, Box and Block Test; EQ-5D-5L, EuroQol 5-Dimension 5-Level; EQ-VAS, EuroQol Visual Analog Scale; FIM, Functional Independence Measure; NHPT, Nine-Hole Peg Test; NRS, numerical rating scale; POD, postoperative day; WCRS, Writer’s Cramp Rating Scale. Interventions: ① Transcutaneous electrical nerve stimulation (TENS); ② fine motor training; ③ vibratory stimulation. Note: The WCRS scores markedly decreased after surgery (Preoperative vs. Initial) but remained unchanged across postoperative assessment points. Higher scores indicate improvement in BBT, EQ-5D-5L, EQ-VAS, Barthel Index, and FIM; lower scores indicate faster performance in the NHPT and reduced severity in the WCRS. —: Not assessed. The preoperative WCRS was assessed by a neurosurgeon, while postoperative measures were assessed by an occupational therapist.

**Figure 2:**
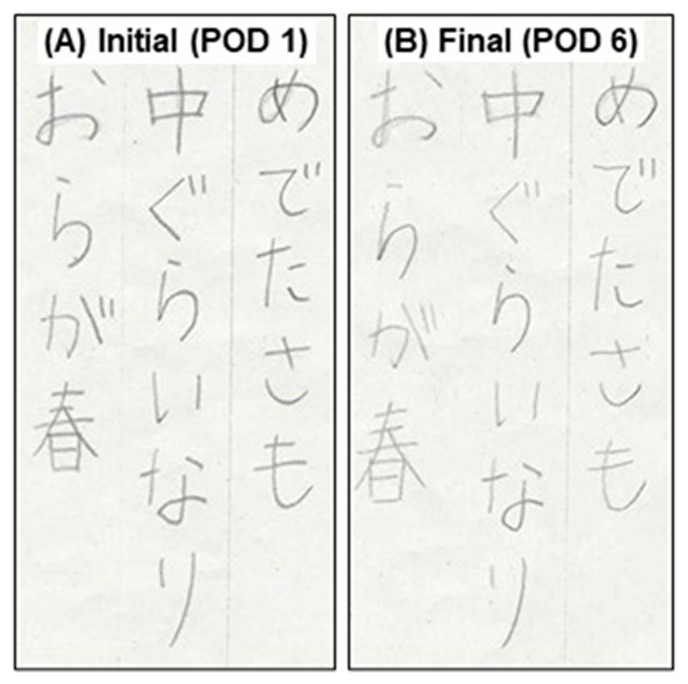
**Handwriting of a fixed 15-character sentence at the initial (A, postoperative day [POD 1]) and final (B, POD 6) assessments; showing a lighter stroke appearance, with intact stroke terminals and stable trajectories**. The sentence reads: “Even as I watch the bright festivities with which others celebrate the New Year, the ‘spring’ that comes to me is no more than a modest, middling sort of good fortune” [めでたさも中ぐらいなりおらが春]. Abbreviations: POD, postoperative day.

Table 1 shows the immediate within-session changes, and short-term changes (from POD 1 to POD 6). Writing time decreased immediately after each intervention and self-reported writing satisfaction (NRS) gradually increased (Table 1). Video 2 confirms the reduction in writing time between POD 1 and 6. No morphological deformation of the stroke terminals was observed, and the trajectory stability showed no wobble. Furthermore, a lighter stroke appearance suggesting reduced pen pressure, was observed on POD 6 compared to POD 1 (Figure 2). Visual comparison between the POD 1 and POD 6 postoperative (Video 2) states demonstrated a visible reduction in the excessive wrist flexion angle during writing. This contrasts with the preoperative video (Video 1), in which severe wrist flexion and co-contraction were prominent. Right-hand dexterity showed an improvement (BBT: 51 to 57 blocks; NHPT: 22.3 s to 20.5 s). The WCRS scores remained stable during the rehabilitation period (POD 1 to POD 6). HRQoL showed an improvement (EQ-5D-5L index: 0.612 to 0.772; EQ-VAS: 40 to 70). Notably, changes in BBT, EQ-5D-5L index, and EQ-VAS exceeded the published minimal detectable change (MDC) or minimal clinically important difference (MCID) thresholds [17,18,19]. Basic activities of daily living were at baseline ceiling levels and remained unchanged.

**Video 2:** **Handwriting performance at the initial postoperative (POD) 1 and final POD 6 assessments**. A comparison between POD 1 and 6 revealed a reduction in writing time and an observable reduction in the wrist flexion angle during writing at the final assessment.

## Discussion

The principal findings were progressive short-term gains and reproducible within-session improvements in writing performance during an intensive multimodal neuromodulatory rehabilitation program after thalamotomy for writer’s cramp. The presence of immediate within-session effects indicated that at least part of the improvement reflected treatment-related mechanisms beyond spontaneous postoperative recovery.

Notably, a lighter stroke appearance was observed on POD 6 compared to POD 1; along with improvements in writing speed and satisfaction. This change likely reflects the mitigation of co-contraction, which enables a lighter and more efficient stroke execution. Thalamotomy resulted in a marked decrease in WCRS scores (movement score: 12 to 4; speed score: 2 to 1), indicating alleviated dystonia severity. However, during the postoperative rehabilitation phase (POD 1 to POD 6), these scores remained unchanged; while kinematic changes and HRQoL improvements were observed. This suggests that while the WCRS successfully captured the surgical benefit; it may lack the sensitivity to detect subtle, immediate gains in writing speed and ease during the acute postoperative phase; which were more effectively captured by time-based and patient-reported measures.

Mechanistically, we hypothesize two distinct processes that may have contributed to the observed outcomes, explicitly acknowledging the speculative nature of this interpretation given the single-case design. First, the immediate within-session gains likely reflect the transient neuromodulatory effects mediated by bottom-up sensory input (TENS and vibratory stimulation), which may temporarily normalize cortical excitability [9,10]. Second, the progressive enhancement across sessions suggests short-term retention or motor learning; potentially facilitated by the execution of top-down task-specific training during these windows of optimized excitability [11]. This distinction offers a biologically coherent framework for interpreting the transient performance boosts vis-à-vis the cumulative functional recovery.

Immediate gains appeared to carry over across sessions. Pre-intervention performance in later sessions did not return to baseline, suggesting a short-term retention that likely contributed to the overall trajectory. Thalamotomy may establish a new neurological baseline, whereas targeted daily rehabilitation may capitalize on heightened plasticity to facilitate functional recovery.

This study had some limitations. First, generalizability is limited by the single-case design. Second, spontaneous postoperative recovery, including the reduction or resolution of postoperative cerebral edema; is a potential confounder [20]. The six-day trends likely reflect both spontaneous and treatment effects; however, immediate within-session changes are unlikely to be explained solely by spontaneous recovery, supporting the direct contribution of therapy. Third, the MDC/MCID values cited for context were derived from non-dystonian populations (such as stroke or general Japanese cohorts) and warrant cautious interpretation. Fourth, writing time was measured in a single trial rather than calculated as the mean of multiple trials; which may limit the reliability of the data. Fifth, the self-directed handwriting practice (daily diary) was not systematically quantified; representing a source of uncontrolled exposure. Sixth, quantitative assessments other than the WCRS were not conducted preoperatively; preventing a direct quantitative comparison for dexterity and HRQoL measures between preoperative and postoperative statuses. Finally, although the patient presented with complex writer’s cramp; we did not quantitatively assess tasks other than writing (e.g., keyboard operation). Because vibratory stimulation was introduced only during later sessions, modality-specific effects could not be isolated.

In conclusion, this case report suggests that a multimodal rehabilitation program integrating TENS, vibratory stimulation, and fine motor training; was associated with immediate within-session gains and progressive short-term improvements in writing performance after thalamotomy for writer’s cramp. These immediate effects support the view that pairing bottom-up neuromodulation with task-specific training may facilitate early postoperative recovery; however, confirmation of efficacy and generalizability requires well-controlled studies with larger samples and longer follow-ups.
